# Sex-Specific Mediation Effects of Workplace Bullying on Associations between Employees’ Weight Status and Psychological Health Impairments

**DOI:** 10.3390/nu13113867

**Published:** 2021-10-29

**Authors:** Hans-Christian Puls, Ricarda Schmidt, Markus Zenger, Hanna Kampling, Johannes Kruse, Elmar Brähler, Anja Hilbert

**Affiliations:** 1 Integrated Research and Treatment Center AdiposityDiseases, Behavioral Medicine Research Unit, Department of Psychosomatic Medicine and Psychotherapy, University of Leipzig Medical Center, 04103 Leipzig, Germany; ricarda.schmidt@medizin.uni-leipzig.de (R.S.); markus.zenger@h2.de (M.Z.); elmar.braehler@medizin.uni-leipzig.de (E.B.); anja.hilbert@medizin.uni-leipzig.de (A.H.); 2 Faculty of Applied Human Studies, University of Applied Sciences Magdeburg and Stendal, 39576 Stendal, Germany; 3 Department of Psychosomatic Medicine and Psychotherapy, Justus Liebig University Giessen, 35390 Giessen, Germany; hanna.kampling@psycho.med.uni-giessen.de (H.K.); johannes.kruse@psycho.med.uni-giessen.de (J.K.); 4 Department of Psychosomatic Medicine and Psychotherapy, University Medical Center of the Johannes Gutenberg University of Mainz, 55131 Mainz, Germany

**Keywords:** weight discrimination, workplace bullying, burnout, sex-specific differences, moderated mediation

## Abstract

Background: Individuals with obesity face weight-related discrimination in many life domains, including workplace bullying, especially in female employees with obesity. However, associations between experiences of workplace bullying and psychological health impairments considering weight status and sex remain unclear. Methods: Within a representative population-based sample of *N* = 1290 employees, self-reported experiences of workplace bullying were examined for variations by weight status and sex. Using path analyses, sex-specific mediation effects of workplace bullying on associations between weight status and work-related psychological health impairments (burnout symptoms, quality of life) were tested. Results: Employees with obesity experienced more workplace bullying than those with normal weight. Workplace bullying was positively associated with psychological health impairments and partially mediated the associations between higher weight status and elevated burnout symptoms and lower quality of life in women, but not in men. Conclusions: The result that more experiences of workplace bullying were, compared with weight status, more strongly associated with work-related psychological health impairments in women, but not in men, uniquely extends evidence on sex-specific effects within weight-related discrimination. Continued efforts by researchers, employers, and policy makers are needed to reduce weight-related discrimination in work settings, eventually increasing employees’ health and job productivity.

## 1. Introduction

Especially in Western societies, a growing number of individuals with obesity (body mass index (BMI) ≥ 30 kg/m^2^ [1]) are at risk of a range of medical and psychological health impairments [2,3]. Although obesity has a complex and multifactorial etiology, it is considered to be the main nutritional disorder, as it ultimately results from an imbalance between caloric intake and caloric expenditure [4]. Aspects that cause additional strain in many life domains (e.g., work settings) of individuals with obesity include weight-related stereotypes (i.e., negative beliefs about a stigmatized group), prejudices (i.e., negative emotions against a stigmatized person), and discrimination (i.e., negative behaviors towards a stigmatized person [5]) such as workplace bullying [6]. The present study aimed to elucidate the intensity of experiences of workplace bullying across the weight range and its associations with psychological health impairments moderated by employees’ sex.

In work settings, weight-related stereotypes describe individuals with obesity as, for example, being less effective, less ambitious, and showing more non-medical absenteeism compared with their co-workers with normal weight [7]. Meta-analytic evidence from experimental studies using simulated employment decisions showed that applicants with obesity, compared with those without obesity, were evaluated more negatively on a range of work-related characteristics, including lower hiring recommendations, lower estimated job success, and less job suitability. Most strikingly, the greatest difference in these simulated attributions to applicants with and without obesity was a lower rating of estimated co-worker desirability for those having obesity [8]. In fact, workers with obesity reported frequent experiences of being bullied or socially isolated by their co-workers [7], which was termed workplace bullying [9]. Within a single observational study on two samples of *N* = 341 student employees and *N* = 528 full-time employees, self-reported experiences of workplace bullying were greater in employees with overweight and obesity than in those with normal weight [10]. Nonetheless, more population-based research considering additional moderators (e.g., age and socioeconomic status) would add to the yet limited evidence regarding experiences of workplace bullying across the weight range [11].

The only available study examining sex-specific effects in workplace bullying across the weight range revealed that women with overweight and obesity reported significantly more experiences of workplace bullying than women with normal weight, while this effect was not found in men, thus indicating a weight-by-sex interaction effect on experiences of workplace bullying [10]. In addition, evidence consistently showed women to be more affected by other forms of weight-related discrimination at the workplace than men, especially regarding recruitment and income [12,13]. For example, in a recent population-based study following-up *N* = 6000 middle and high school seniors over four years after graduation, young women with overweight or obesity had less job chances and earned less than those with normal weight, while the opposite effect was found in men [14]. It may be concluded that women experience stronger weight-related discrimination at the workplace, including workplace bullying, than men.

General experiences of weight-related discrimination were associated with a range of medical and psychological health impairments, including eating disturbances (e.g., high-caloric food intake, binge-eating, or emotional eating; [15]). Specific psychological correlates of workplace bullying included elevated symptoms of emotional exhaustion and lower quality of life, both in workers across the weight range [16,17] and in workers with overweight or obesity compared with those with normal weight [18,19]. Further, in the study by Sliter et al. [10], experiences of workplace bullying partially mediated the association between weight status and job withdrawal, with the latter being associated with emotional exhaustion. Alongside depersonalization and reduced perceived accomplishment at work, emotional exhaustion represents a key symptom of the burnout syndrome [20], which itself is known to be an important health issue in work settings [21]. Notably, in sex-specific analyses, experiences of workplace bullying were more strongly associated with weight status in women than in men, and linked to job withdrawal in men, but not in women [10].

Research has only begun to elucidate the prevalence of experiences of workplace bullying and their sex-specific associations with psychological health impairments. Thus, the present study aimed to, firstly, describe the intensity of experiences of workplace bullying in a large population-based sample as a function of employees’ weight status and sex. We expected more experiences of workplace bullying in individuals with overweight and obesity than with normal weight and, within participants with overweight or obesity, in women than in men. Secondly, weight status and workplace bullying were evaluated regarding their relative explanatory power for work-related psychological health impairments, hypothesizing a mediating effect of workplace bullying on the association between higher weight status and elevated burnout symptoms as well as lower quality of life. Thirdly, we evaluated whether this mediating effect would be moderated by sex (i.e., evident in women, but not in men).

## 2. Materials and Methods

### 2.1. Recruitment and Sample

The present study’s data were derived from a 2012 representative survey of the German population with the assistance of the Independent Service for Surveys, Methods, and Analyses (USUMA Berlin). The sampling procedure, as a common and standardized procedure in German representative survey designs [22], included three stages of randomized selection, and thus yielded in a sample representative for the German general population. First, 258 representative sampling areas were selected out of a total of 53,000 areas from all parts of Germany, stratified according to counties and number of inhabitants [23]. Second, 4386 addresses were selected using a random-route-assisted procedure and, third, one randomly selected household member per address was personally contacted and supposed to respond in person for study participation. The survey was approved by the Ethics Committee of the University of Leipzig (Approval No. 072-11-07032011) and followed the ethical guidelines of the International Code of Marketing and Social Research Practice by the International Chamber of Commerce and the European Society for Opinion and Marketing Research. Oral informed assent and consent was obtained from the participants for ≥18 years and, for participants <18 years, informed consent was obtained from the parents, which is common in survey research in Germany. Following a face-to-face explanation of the study objectives, the procedure, and data protection, each participant received a questionnaire, which he or she completed himself or herself in the presence of the interviewer.

Of the 2555 participants who responded to the initial contact (response rate 58.3%), *n* = 1335 (52.3%) participants were employed at the time of the survey and provided data on experiences of workplace bullying derived from the “Intensity of Bullying Coming from Co-Workers” scale (MOB-K, see below [24]). As weight status was a central study variable, we omitted *n* = 20 participants owing to missing data on weight and height, and we further omitted *n* = 25 participants with underweight (BMI ≤ 18.5 kg/m^2^) owing to their small number, finally resulting in a study sample of *N* = 1290 participants (50.5% out of 2555 eligible individuals; 29.4% out of 4386 initially contacted individuals).

### 2.2. Measures

*Workplace bullying.* Experiences of workplace bullying were assessed using the sum score of the MOB-K [24]. The sum score is derived from four items addressing different aspects of workplace bullying (i.e., social isolation, bullying, defamatory statements, and overall quality of interaction), which are rated on a four-point Likert scale (1 = *not at all* to 4 = *very much*). Higher scores indicate higher intensity of experiences of workplace bullying. In the present study, the MOB-K showed good internal consistency (Cronbach’s α = 0.83).

*Psychological health impairments.* To depict work-related psychological health impairments, we focused on work-related symptoms of exhaustion, distress, dysfunctional cognitions, and feelings of decreased efficacy and motivation, all associated with the burnout syndrome [20], and assessed by the “Burnout Screening Scale II” (BOSS II [25]). With 30 items rated on a six-point Likert scale (0 = *not at all* to 5 = *very much*), the BOSS II assesses physical, cognitive, and emotional symptoms occurring within the last seven days. Items are accumulated to a mean score, with higher scores indicating higher impairment. Quality of life was assessed using the sum score of the “EuroQoL 5 Item-index” (EQ-5D [26,27]). Five items addressing impairments in mobility, self-care, usual activities, pain/discomfort, and anxiety/depression are rated on a five-point Likert scale (1 = *not at all* to 5 = *very much*) and accumulated to a sum score, with higher scores indicating a higher quality of life. BOSS II and EQ-5D showed good to high internal consistency in the present study (Cronbach’s α = 0.83 and 0.96, respectively).

*Weight and socioeconomic status.* Participants were categorized into weight status groups according to their BMI (kg/m^2^), specifically, normal weight (18.5 ≤ BMI < 25.0 kg/m^2^), overweight (25.0 ≤ BMI < 30.0 kg/m^2^), and obesity (BMI ≥ 30.0 kg/m^2^), based on self-reported weight and height. Participants were further categorized into low, medium, and high socioeconomic status (SES) as derived from a modified Winkler Index, which accumulates information about the highest educational degree, professional degree, current profession, and household net income [28]. Sociodemographic and clinical characteristics of the total sample, sex-specific subsamples, and across the weight range are depicted in Table 1 and Supplementary Table S1, respectively.

### 2.3. Data Analytics Plan

First, to rule out possible common method variance bias due to the self-report nature of our data [29,30], we applied Harman’s single-factor test on all measures prior to data analysis [31]. Based on an eight-factor solution explaining 65.5% of variance, with the first factor accounting for 36.4%, the bias of common method variance in the present data was assumed to be low [30]. Using univariate analyses of covariance (ANCOVA; IBM^®^ SPSS version 25.0; Chicago, IL, USA) on the MOB-K sum score by weight status, sex, and their interaction, weight- and sex-specific differences were identified, controlling for age and SES. Although all dependent variables deviated from normal distribution, which is common in large samples [32], ANCOVA was used because of its robustness against non-normality [33] and the large sample being selected with a high degree of randomization. However, all analyses were repeated using non-parametric methods, specifically the Kruskal–Wallis test, and their results will be reported if deviating from parametric analyses. Post-hoc analyses included unpaired t-tests, applying Bonferroni-corrected significance levels. Cohen’s *d* was used as an effect size measure, representing small (≥0.2), medium (≥0.5), and large (≥0.8) effects [34].

Second, using path analysis (IBM^®^ SPSS AMOS^®^ version 25.0; Chicago, IL, USA), we tested the possible mediating effect of experiences of workplace bullying on the association between weight status and psychological health impairments. As a first step, direct effects of weight status on psychological health impairments (BOSS II and EQ-5D) were examined for the total sample (Model 1), controlling for socioeconomic variables (sex, age, and SES). Subsequently, the indirect effects of weight status on mental health variables, possibly mediated by the experiences of workplace bullying (MOB-K sum score), were examined (Model 2).

Third, possible sex-specific mediation effects (i.e., moderated mediation [35,36]) of the experiences of workplace bullying on the associations between weight status and psychological health impairments were examined using the SPSS PROCESS macro version 3.5 [36], which utilizes bootstrapping to assess direct and indirect effects of variables while maximizing power and minimizing concerns about non-normality. Furthermore, 95% confidence intervals were resampled 5000 times for each analysis to test the significance of the indirect effects [37]. To additionally confirm and depict sex-specific differences regarding the explanatory power of weight status and experiences of workplace bullying for psychological health impairments, the final model from the path analysis (Model 2) was applied to subsamples of women (Model 3) and men (Model 4) separately. For path analyses, the following indices were determined for the evaluation of model fit: χ^2^ test statistics; the minimum discrepancy, divided by its degrees of freedom (CMIN/DF); the comparative-fit index (CFI); the Tucker–Lewis index (TLI); the normed-fit index (NFI); and the root mean square error of approximation (RMSEA). Good model fit is indicated by non-significant χ^2^ statistics; CMIN/DF < 2; CFI, TLI, and NFI > 0.97; and RMSEA < 0.08 [38]. Standardized regression weights were interpreted as indicative of small (≤0.30), medium (between 0.30 and 0.50), or large (>0.50) effects [34]. All analyses used a two-tailed α < 0.05 as the significance level.

## 3. Results

### 3.1. Weight- and Sex-Specific Differences in Experiences of Workplace Bullying

Regarding experiences of workplace bullying, we found a significant main effect of weight status, *F* (2, 1276) = 4.24, *p* = 0.02 (*d* = 0.16), but no significant main effect of sex, *F* (1, 1276) = 1.80, *p* = 0.18 (*d* = 0.07), and no significant interaction effect of weight status x sex, *F* (2, 1276) = 0.73, *p* = 0.48 (*d* = 0.07). Post-hoc analyses using a Bonferroni-corrected significance level of 0.016 revealed that, compared with participants with normal weight, levels of experiences of workplace bullying were significantly greater in participants with obesity (*p* = 0.01, *d* = 0.34). The level of experiences of workplace bullying did not significantly differ between participants with normal weight and those with overweight (*p* = 0.04, *d* = 0.12) and between those with overweight and those with obesity (*p* = 0.15, *d* = 0.17).

### 3.2. Associations of Weight Status and Experiences of Workplace Bullying with Psychological Health Impairments

**Figure 1:**
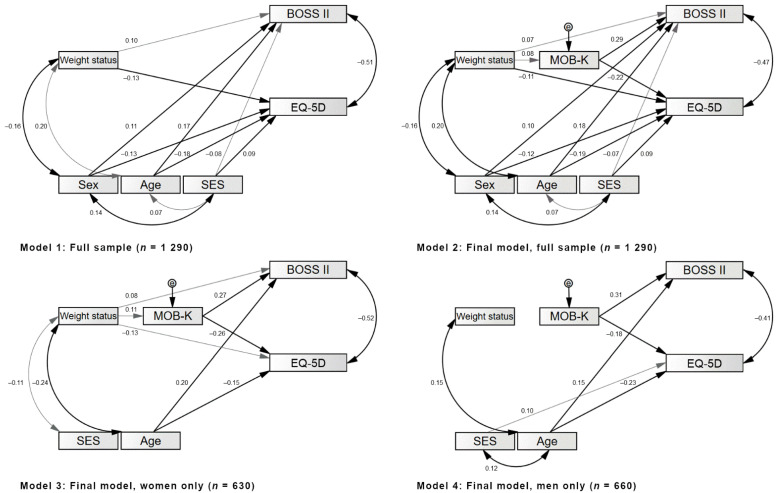
Models 1 to 4: Direct and indirect effects of weight status and workplace bullying on psychological health impairments while controlling for sociodemographic variables. Notes: Standardized regression weights are depicted. Only significant associations are depicted. Different significance levels are depicted with black lines (*p* < 0.001) or gray lines (*p* < 0.05). Burnout symptoms measured by Burnout Screening Scales II. Quality of life measured by Euro-Qol Quality of Life Index. SES = socioeconomic status.

The results of the path analyses are presented in Figure 1 for all models. All models showed good model fit, as depicted in Table 2. In Model 1, a significant direct effect of a higher weight status on elevated burnout symptoms (β = 0.10) and a lower quality of life (β = −0.13) was found. In Model 2, a higher weight status was significantly associated with more experiences of workplace bullying (β = 0.08), with the latter being associated with higher burnout symptoms (β = 0.29) and with a lower quality of life (β = −0.22), showing the indirect effect of weight status on psychological health impairments. In this final model, a higher weight status was still associated with elevated burnout symptoms (β = 0.07) and with a lower quality of life (β = −0.11), thus indicating partial mediation effects of experiences of workplace bullying on the association between higher weight status and elevated burnout symptoms and a lower quality of life.

In sex-specific analyses, we found that weight status was positively associated with experiences of workplace bullying for women (Model 3, β = 0.12), but not for men (Model 4, β = 0.06), thus alone suggesting no mediational effect of experiences of workplace bullying in men. In women, but not in men, a higher weight status was associated with elevated burnout symptoms (β = 0.08) and with a lower quality of life (β = −0.13). Utilizing the PROCESS macro [37], we affirmatively found the mediational effect of experiences of workplace bullying to be moderated by sex. Specifically, while we found direct effects of weight status on burnout symptoms (β = 0.09) and quality of life (β = −0.36) for women and men, indirect effects (i.e., mediated by experiences of workplace bullying) were only evident for women, but not for men, regarding both burnout symptoms (β = 0.03) and quality of life (β = 0.07). Thus, specifically for women, experiences of workplace bullying partially mediated the association between a higher weight status and elevated burnout symptoms and a lower quality of life.

## 4. Discussion

Derived from a large population-based sample, the present study showed that, in line with our hypotheses and previous research [10], individuals with obesity encounter more frequent experiences of workplace bullying than individuals with normal weight. Experiences of workplace bullying were significantly associated with psychological health impairments and emerged as a partial mediator on the associations between higher body weight and elevated burnout symptoms and a lower quality of life, with this effect being particularly evident in women, but not in men. Based on data from real-life employment settings in Germany, the present results extend previous experimental research on sex-specific weight-related discrimination at the workplace, which mainly focused on recruitment and income [7,8,10,11,12,13] by experiences of workplace bullying.

In line with our hypotheses and previous research [16,17,18,19,39], more experiences of workplace bullying were associated with elevated burnout symptoms and lower quality of life. These associations were substantially stronger than associations between a higher weight status and a greater risk for psychological health impairments. Experiences of workplace bullying partially mediated the association between a higher weight status and elevated burnout symptoms in the total sample, in line with the mediation effect found by Sliter et al. [10] of workplace bullying on the association between weight status and job withdrawal. Further, experiences of workplace bullying partially mediated the association between a higher weight status and a lower quality of life, altogether indicating that a higher weight status alone is linked to psychological health impairments, while additionally experiencing workplace bullying is associated with a further increased health risk. However, because the associations between weight status and psychological health impairments were only reduced to a small extent (Δβ’s ranging from 0.02 to 0.03) after including experiences of workplace bullying, the mediation effects may not be necessarily clinically relevant. Importantly, owing to design, the present results depict cross-sectional associations, leaving unclear the causal mechanisms between weight status, experiences of workplace bullying, burnout symptoms, and quality of life. Thus, all mediation effects must be interpreted considering this important limitation.

Differences in experiences of workplace bullying between women and men across the weight range were shown within sex-specific subsamples in the path analyses, in which a higher weight status was significantly associated with more frequent experiences of workplace bullying in women (Model 3), but not in men (Model 4), replicating findings by Sliter et al. [10] within a larger and representative sample from the general population. However, we found no weight status-by-sex interaction effect on the level of experiences of workplace bullying in the univariate statistics, as previously revealed by Sliter et al. [9]. In the present study, only descriptively, experiences of workplace bullying tended to be increased in women with obesity compared with men with obesity (*p* = 0.28, *d* = 0.24). In contrast to Sliter et al. [10], who showed that men with underweight were more likely to experience workplace bullying than women with underweight, in the present study, we excluded participants with underweight owing to the small sample size (*n* = 25, 1.9%), which may explain the lack of the interaction effect in the present study.

In the present study, derived from both the path analyses and the SPSS PROCESS macro [37], partial mediation effects found in the total sample were evident for women, but not for men, indicating sex-specific differences regarding the adverse psychological health correlates of workplace bullying. In women, but not in men, a higher weight status had direct and indirect effects on elevated burnout symptoms and a lower quality of life, with these associations being partially mediated by experiences of workplace bullying. Thus, while women with obesity were at risk for elevated burnout symptoms and a lower quality of life, those who additionally experienced workplace bullying showed an even increased health risk. Considering the deleterious impact of burnout symptoms on a range of adverse medical conditions (e.g., type 2 diabetes and coronary heart disease), mental health (e.g., insomnia and depressive symptoms), and occupational outcomes (e.g., low job satisfaction and absenteeism) [40], and given that future longitudinal studies could replicate the present findings, employers and policy makers might further address workplace bullying, potentially preventing medical and psychological health impairments for workers and associated productivity losses for organizations [20,41].

Strengths of the present study comprise the use of established measures to depict work-related psychological health impairments within a large sample from the general population, which was representative for the German population both in terms of sex ratio and SES [42,43], and included participants from real-life employment settings, as previously recommended [7]. However, the sample was not generally representative for the prevalence of obesity, as obesity was underrepresented in the present sample (6.4%) as compared with the German general population (23.6% [44]), which might be explained by participants’ impression management, specifically by an underreporting of body weight, which was previously found in individuals with BMI ≥ 20 kg/m^2^, and an overreporting in those with BMI < 20 kg/m^2^ [45]. As this bias in self-reported BMI is of high concern for large epidemiological studies within obesity-related research, studies investigating novel methods of large-scale measurement of weight and height are urgently needed (e.g., corrective equations or BMI self-selection [46,47]). A major limitation of the present study is that all analyses used cross-sectional data, leaving unclear the causal mechanisms between body weight, experiences of workplace bullying, and work-related psychological health impairments. Ideally, studies from non-Western countries might replicate our procedures to allow generalization of the results. Finally, as no specific information on participants’ occupation was provided, it was not possible to systematically examine the effects considering other possibly relevant work-related variables, such as the number of colleagues or type of work sector.

Most importantly, future research should explore the prospective associations between body weight, experiences of workplace bullying, and psychological health impairments to elucidate their causal relationships. The present result that individuals with obesity, compared with those with normal weight, encounter more frequent experiences of workplace bullying, with the latter being more strongly linked to work-related psychological health impairments than the weight status, suggests continued efforts by researchers and employers to address and eventually reduce experiences of workplace bullying (e.g., by incorporating information on bullying into existing education platforms within the workplace [48,49]). As our study did not focus on discrimination owing to ethnicity or race, future studies should take discriminated features other than weight and sex (e.g., low SES and mental disorders [50,51]) into account when exploring the adverse effects of stigmatization. Our results suggest that, especially in women with obesity, who are at particular risk of experiencing weight-related discrimination in various life domains [52], different discriminated features (i.e., weight status and sex) may add up to multiple layers of stigmatization. Thus, especially in women with obesity, reducing experiences of workplace bullying will likely yield a decreased psychological burden of employees, and might strengthen their health status and lower their odds of adverse occupational outcomes (e.g., job withdrawal or absenteeism).

## Figures and Tables

**Table 1:** Sociodemographic and clinical variables for the total sample and sex-specific subsamples, including sex-specific differences using *t*-tests for continuous and χ^2^-tests for categorical variables.

Variable	Total Sample (*n* = 1290)	Women (*n* = 630, 48.8%)	Men (*n* = 660, 51.2%)	Sex-Specific Differences		
	*M (SD)*	*M (SD)*	*M (SD)*	*T*	*df*	*p*
Age (years)	43.06 (12.48)	43.22 (12.21)	42.91 (12.74)	−0.45	1288	0.654
BMI (kg/m^2^)	24.86 (3.48)	24.26 (3.65)	25.44 (3.20)	6.19	1288	<0.001
	*n* (%)	*n* (%)	*n* (%)	χ^2^	*df*	*p*
Weight status				70.82	2, 1290	<0.001
Normal weight (18.5 ≤ BMI < 25.0 kg/m^2^)	714 (55.3)	417 (66.2)	297 (45.0)			
Overweight (25.0 ≤ BMI < 30.0 kg/m^2^)	494 (38.3)	168 (26.7)	326 (49.4)			
Obesity (BMI ≥ 30.0 kg/m^2^)	82 (6.4)	45 (7.1)	37 (5.6)			
Socioeconomic status (SES)				86.35	2, 1277	<0.001
Low	312 (24.2)	84 (13.3)	228 (34.5)			
Middle	770 (59.7)	447 (71.0)	323 (49.0)			
High	195 (15.1)	92 (14.6)	103 (15.6)			
German Citizenship				9.89	1, 1290	<0.01
Yes	1226 (95.0)	611 (97.0)	615 (93.2)			
No	64 (5.0)	19 (3.0)	45 (6.8)			
	*M (SD)*	*M (SD)*	*M (SD)*	*T*	*df*	*p*
Workplace bullying (MOB-K)	4.76 (1.63)	4.76 (1.61)	4.76 (1.64)	−0.07	1288	0.948
Burnout symptoms (BOSS-II)	0.50 (0.60)	0.54 (0.63)	0.44 (0.57)	−2.93	1286	<0.01
Quality of life (EQ-5D)	24.08 (1.65)	23.92 (1.80)	24.24 (1.49)	3.50	1288	<0.001

Notes. BMI = body mass index (kg/m^2^); SES = socioeconomic status (Lange et al., 2007); MOB-K = Intensity of Bullying Coming from Co-Workers scale sum score (4–16*, less favorable scores are asterisked; Pfaff, Bentz, & Brähler, 2007); BOSS II = Burnout Screening Scale II mean score (0–5*; Hagemann & Geuenich, 2009); EQ-5D = EuroQoL 5 Item-index sum score (5*–25; Hinz, Kohlmann, Stöbel-Richter, Zenger, & Brähler, 2014; Janssen et al., 2013). Statistical analyses used a two-tailed α < 0.05 significance level. *—less favorable scores are asterisked.

**Table 2:** Path analysis on the mediating effect of workplace bullying on associations between weight status and psychological health impairments: model fit indices.

Model Fit Indices	*n*	χ^2^	*df*	*p*	CMIN/DF	CFI	RMSEA	TLI	NFI
1: Weight status only	1290	0.201	1	0.654	0.201	1.000	0.000	1.025	1.000
2: Final Model, total sample	1290	0.889	3	0.828	0.296	1.000	0.000	1.025	0.999
3: Final Model, women only	630	1.232	1	0.267	1.232	1.000	0.019	0.989	0.997
4: Final Model, men only	660	0.116	1	0.734	0.116	1.000	0.000	1.061	1.000

Notes. *df* = degrees of freedom; BMI = body mass index (kg/m^2^); CMIN/DF = minimum discrepancy, divided by degrees of freedom; CFI = comparative-fit index; RMSEA = root mean square error of approximation; TLI = Tucker–Lewis index; NFI = normed fit index.

## Data Availability

Research data are not shared.

## Supplementary Materials

The following are available online at https://www.mdpi.com/article/10.3390/nu13113867/s1, Supplementary Table S1: Sex-specific experiences of workplace bullying and psychological health impairments across the weight status: Mean values and standard deviations.
