# Supplementary material for: Sex-Specific Mediation Effects of Workplace Bullying on Associations between Employees’ Weight Status and Psychological Health Impairments
Source: Nutrients. 2021 Oct 29;13(11):3867. doi: 10.3390/nu13113867 (PMC8625383; doi:10.3390/nu13113867)
Supplement: Supplementary file 1 [file nutrients-13-03867-s001.zip › nutrients-1420799-supplementary.pdf]

Sex-specific mediation effects of workplace bullying on associations between employees' weight status and psychological health impairments

Supplementary Materials

Supplementary Table S1

Sex-specific experiences of workplace bullying and psychological health impairments across the weight status: Mean values and standard deviations

Variable	Total sample (n = 1 290)			Women (n = 630, 48.8%)			Men (n = 660, 51.2%)		
Weight status	NW	OW	OB	NW	OW	OB	NW	OW	OB
n	714	494	82	417	168	45	297	326	37
Workplace bullying (MOB-K)	4.65 (1.43)	4.85 (1.80)	5.16 (2.01)	4.65 (1.42)	4.87 (1.84)	5.38 (2.20)	4.65 (1.45)	4.84 (1.79)	4.89 (1.74)
Burnout symptoms (BOSS-II)	0.45 (0.57)	0.49 (0.59)	0.84 (0.80)	0.49 (0.61)	0.55 (0.60)	0.99 (0.80)	0.40 (0.51)	0.46 (0.59)	0.66 (0.77)
Quality of life (EQ-5D)	24.23 (1.49)	24.08 (1.64)	22.88 (2.43)	24.10 (1.63)	23.87 (0.55)	22.42 (2.55)	24.40 (1.24)	24.19 (1.56)	23.43 (2.17)

Notes. Weight status groups, according to the BMI (body mass index, kg/m²): NW = Normal weight (18.5 ≤ BMI < 25.0 kg/m²), OW = Overweight (25.0 ≤ BMI < 30.0 kg/m²), OB = Obesity (BMI ≥ 30.0 kg/m²); MOB-K = Intensity of Bullying Coming from Co-Workers scale sum score (4 – 16*, less favorable scores are asterisked; Pfaff, Bentz & Brähler, 2007); BOSS II = Burnout Screening Scale II mean score (0 – 5*; Hagemann & Geuenich, 2009); EQ-5D = EuroQoL 5 Item-index sum score (5* – 25; Hinz, Kohlmann, Stöbel-Richter, Zenger, & Brähler, 2014; Janssen et al., 2013).
